# Retraction: CXCL6 promotes renal interstitial fibrosis in diabetic nephropathy by activating JAK/STAT3 signaling pathway

**DOI:** 10.3389/fphar.2024.1530358

**Published:** 2024-12-05

**Authors:** Frontiers Editorial Office

The journal retracts the 25 March 2019 article cited above.

Following publication, concerns were raised regarding the integrity of the images in the published figures. Image duplication concerns were identified in Figure 6C. The authors failed to provide a satisfactory explanation during the investigation, which was conducted in accordance with Frontiers’ policies. As a result, the data and conclusions of the article have been deemed unreliable and the article has been retracted.

This retraction was approved by the Chief Editors of Frontiers in Pharmacology and the Chief Executive Editor of Frontiers. The authors agree to this retraction.

